# Conveyor CVD to high-quality and productivity of large-area graphene and its potentiality

**DOI:** 10.1186/s40580-024-00439-0

**Published:** 2024-08-14

**Authors:** Dong Yun Lee, Jungtae Nam, Gil Yong Lee, Imbok Lee, A-Rang Jang, Keun Soo Kim

**Affiliations:** 1 Department of Physics and Graphene Research Institute, Sejong University, Seoul, 05006 Republic of Korea; 2 Division of Electrical, Electronic and Control Engineering, Kongju National University, Cheonan-si, Chungcheongnam-do 31080 Republic of Korea

**Keywords:** Graphene, Synthesis, Doping, Chemical vapor deposition, Conveyor, Productivity, Gas sensor

## Abstract

The mass production of high-quality graphene is required for industrial application as a future electronic material. However, the chemical vapor deposition (CVD) systems previously studied for graphene production face bottlenecks in terms of quality, speed, and reproducibility. Herein, we report a novel conveyor CVD system that enables rapid graphene synthesis using liquid precursors. Pristine and nitrogen-doped graphene samples of a size comparable to a smartphone (15 cm × 5 cm) are successfully synthesized at temperatures of 900, 950, and 1000 °C using butane and pyridine, respectively. Raman spectroscopy allows optimization of the rapid-synthesis conditions to achieve uniformity and high quality. By conducting compositional analysis via X-ray photoelectron spectroscopy as well as electrical characterization, it is confirmed that graphene synthesis and nitrogen doping degree can be adjusted by varying the synthesis conditions. Testing the corresponding graphene samples as gas-sensor channels for NH_3_ and NO_2_ and evaluating their response characteristics show that the gas sensors exhibit polar characteristics in terms of gas adsorption and desorption depending on the type of gas, with contrasting characteristics depending on the presence or absence of nitrogen doping; nitrogen-doped graphene exhibits superior gas-sensing sensitivity and response speed compared with pristine graphene.

**Supplementary Information:**

The online version contains supplementary material available at 10.1186/s40580-024-00439-0.

## Introduction

Since the discovery of graphene [1–3], there has been continuous research on the synthesis of large-area graphene [4, 5]. Conventional batch-type methods for graphene synthesis can be segmented into the following processes: heating, annealing of catalyst metal surface, graphene growth, and cooling; each process is executed independently [6, 7]. However, these methods, despite yielding satisfactory graphene at the laboratory level, are not competitive in terms of future industrial applications owing to the slow production speed. To overcome these bottlenecks, we focused on improving conventional CVD methods, including both systems and recipes.

The roll-to-roll CVD system has been consistently reported for scaling up graphene production [8–10]. In this process, a copper foil roll, which acts as the catalyst metal, is transported within a chamber filled with hydrocarbon gas; a thin carbon film is deposited on the copper surface as it moves to the high-temperature zone. The catalyst metal is continuously moved into and out of the CVD reaction zone, allowing the sequential execution of heating, surface annealing and reduction, growth, and cooling. This process enables the synthesis of large-area graphene in a short time. However, the individual processes cannot be controlled independently. Furthermore, when the metal foil moves continuously, it is exposed to the carbon precursor at a lower temperature than the target temperature in areas far from the reaction zone, resulting in the deposition of an amorphous carbon film. Additionally, the mechanical forces applied to the metal foil during its transportation between the high- and low-temperature zones cause alternating tensile and compressive stresses, creating defects in the graphene [8, 9]. As a result, synthesizing high-quality materials in this system is challenging. Furthermore, because graphene is synthesized on a metal catalyst roll, only flexible substrates must be used, greatly limiting the substrate scope.

Efforts have been made to overcome these challenges, such as mechanically separating the annealing and growth zones [11, 12], or attempting to maintain consistent tension by moving the foil vertically [10]. However, the quality of graphene synthesized has not improved sufficiently. Although numerous studies have reported on the mass production of high-quality large-area graphene, challenges such as the formation of graphene defects and nonuniformities such as adlayers still persist.

In addition, for the practical application of graphene, research on property modulation through doping is crucial. Various methods have been employed for the synthesis of doped graphene, including the plasma post-treatment of pristine graphene and the CVD of dopant-containing carbon precursors. However, the former is prone to a relatively high level of defects and requires multiple processes [13], whereas the latter results in nonuniform doping [14, 15]. Furthermore, research on the mass production of doped graphene has not yet been conducted.

Therefore, the development and optimization of systems and processes that overcome the drawbacks of the existing roll-to-roll CVD system are crucial for the mass production of high-quality graphene with desired properties, which in turn is key for the industrialization of graphene.

**Fig. 1:**
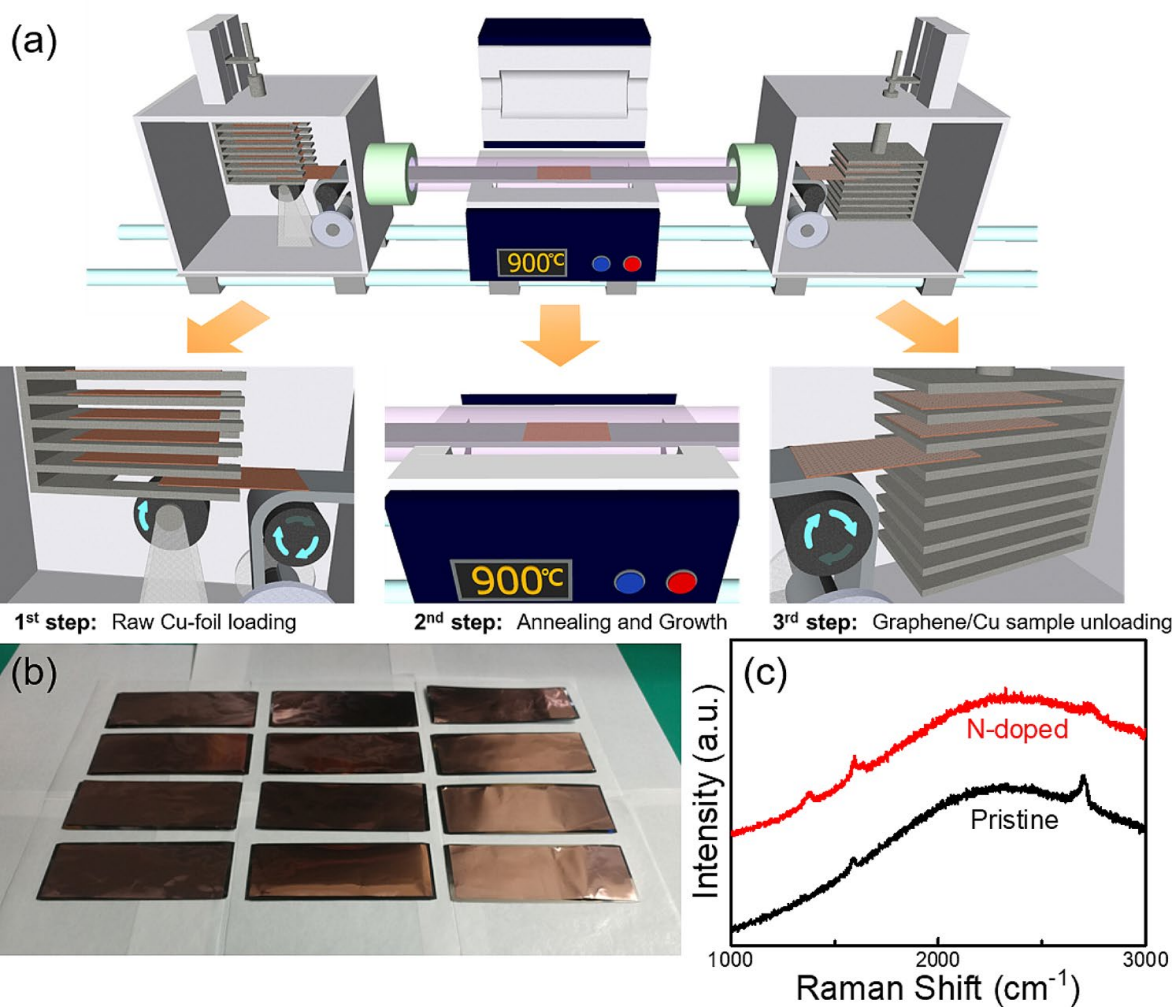
(**a**) Schematic of conveyor CVD system. (**b**) Photograph of twelve as-synthesized graphene samples. (**c**) Raman spectra of as-grown pristine and n-doped graphene on copper foil synthesized at 900 ℃ and 1000 ℃, respectively

In this study, to overcome the drawbacks of the roll-to-roll CVD system, we developed a novel conveyor-type CVD system and optimized its mass-production process (Fig. 1a). As mentioned earlier, conventional batch-type CVD allows high-quality graphene synthesis but is challenging for mass production, whereas roll-to-roll CVD allows mass production but suffers from insufficient quality. The conveyor system retains the advantages of independent process control from segment-based batch-type CVD and continuous production from roll-to-roll CVD, while eliminating the respective drawbacks of low productivity and reduced quality caused by decreased process control flexibility. In our system, while the desired temperature is maintained at the reaction zone for annealing and growth, the substrate prepared in the cartridge sample holder is transported to the reaction zone by a conveyor belt. The process gas is then toggled according to each process segment, completing annealing and growth. After growth, the substrate is transported to the opposite cartridge holder. Importantly, only the carbon-containing precursor is injected during the synthesis, whereas the residual carbon gas is removed during the other processes. This iterative method enables the continuous synthesis of high-quality graphene. Furthermore, it is versatile, allowing for the use of various substrates, such as flexible substrates, rigid wafers, metal foams, and powders (Fig. 1b, Figure S1).

Various carbon sources of all phases have been utilized as graphene precursors, with the growth time and temperature conditions varying significantly depending on their phase [16]. Dehydrogenation of the carbon source is particularly crucial in determining the growth time [17]. For example, traditional CVD with methane gas, which is common for graphene synthesis, requires a prolonged synthesis time of over 10 min at temperatures around 1000 ℃ owing to the high C–H bonding energy [4, 5, 18–20]. Therefore, to achieve mass production of graphene, it is essential to identify suitable carbon precursors and synthesis conditions that allow rapid synthesis. We previously reported the high-speed synthesis of pristine graphene using liquefied petroleum gas (LPG) [21] and nitrogen-doped graphene using pyridine, a nitrogen-containing organic solvent [22]. Based on prior research, we applied these methods to our conventional CVD system, utilizing pure liquefied butane for pristine graphene synthesis and pyridine for N-doped graphene synthesis; consequently, our conveyor CVD system achieved the desired quality of graphene with high productivity and reproducibility. Furthermore, we used the N-doped graphene to enhance the properties of other 2D materials [23]. We optimized the synthesis conditions, namely the growth temperature and time, for the mass production of pristine and N-doped graphene. Finally, we demonstrated the potential application of these pristine and doped samples as gas sensors for various applications.

## Experiment

### Graphene synthesis

Pristine and nitrogen-doped graphene were synthesized using pure butane (99% purity, n-Butane; Sigma-Aldrich) and pyridine (99.8% purity, anhydrous; Sigma-Aldrich), respectively, on a copper foil (99.8% purity, 25 μm thickness; Alfa Aesar) using the conveyor CVD system; various high-speed synthesis conditions were investigated to determine the optimal conditions for the desired quality or dopant content of graphene. In particular, pyridine has a structure wherein one methine group (= CH−) in benzene is replaced by a nitrogen atom (= N−). We aimed to synthesize graphitic N bonds within the graphene lattice by substituting carbon with nitrogen. However, referred to as pyridinic N and pyrrolic N bonds, were also synthesized. These bonds refer to the nitrogen atoms contributing to the pi system with one and two p-electrons respectively, resulting in p-type graphene. Our objective was to determine the optimized conditions for synthesizing graphitic N bonds that make n-type graphene [24, 25]. The sample was heat treated at the target temperatures for a constant 1 min duration of hydrogen treatment at 50 sccm and 380 mTorr. After heat treatment, pristine graphene synthesis using butane (3 sccm; 170 mTorr) or nitrogen-doped graphene synthesis using pyridine (100 mTorr) was conducted over various synthesis times (30, 45, 60, and 90 s) to optimize the synthesis time. Twelve substrates, each sized at 15 cm × 5 cm, were transported from the cartridge holder and subjected to 1 min of heat treatment (Figure S3) and 1 min of growth, resulting in the synthesis of 12 samples in 1 h (Fig. 1b). A supplementary video related to this process can be found at 10.1186/s40580-024-00439-0. The synthesized graphene, supported with a poly methyl methacrylate (PMMA) layer on the top surface, was spin-coated and electrochemically delaminated to separate it from the copper foil [26–28], then transferred onto a SiO_2_ substrate for evaluation of its optical and electrical characteristics, among other properties.

### Characterization

The graphene samples synthesized on copper foil under various conditions were transferred onto 300 nm SiO_2_ substrates for Raman spectroscopy, which was performed on 30 points for each sample, both before and after transfer, using the Renishaw inVia Raman microscope with a 514-nm laser. Additionally, the graphene samples on gold film deposited SiO_2_ substrates were subjected to X-ray photoelectron spectroscopy (XPS) to analyze their elemental compositions and bonding structures. The graphene samples transferred onto 300 nm SiO_2_ substrates were then used to fabricate back-gate field-effect transistors (FETs) with 10 μm × 10 μm graphene channels. These devices were loaded into a vacuum chamber (pressure below 3 mTorr) and interfaced with a source measure unit (SMU; Keithley 236 & 237) using a LabVIEW program. The electrical response characteristics were measured as functions of the gate voltage and source-drain current. To demonstrate the potential applications of the graphene samples, both pristine and nitrogen-doped samples were exposed to NH_3_ and NO_2_, and their electrical characteristics were measured. The gas sensitivity was evaluated using the response properties calculated from the resistance and current changes in each sample channel as a function of gas type, gas concentration, and exposure time.

## Result and discussion

### Raman Spectroscopy

Figure 1a shows the conveyor CVD system process. The substrate was first loaded into the left cartridge and placed on the copper metal foil; the foil acted as a conveyor belt and transported the cartridge to the center of the furnace, where the substrate underwent heat annealing and growth. It was then transported to the right cartridge. Simultaneously, during transport of the substrate from the loading chamber to the growth area, the temperature increased rapidly from room temperature. As reaction with a carbon source during transport can cause the deposition of amorphous carbon, synthesis gas was not supplied, and only hydrogen was supplied for reduction. The furnace was operated at various target temperatures (850, 900, 950, and 1000 ℃) for a 1 min hydrogen treatment to optimize the heat treatment temperature. The heat treatment time was varied to find the minimum temperature for rapid synthesis (Figure S2).

Figure 1c shows the Raman spectra of pristine and nitrogen-doped graphene synthesized on copper at 900 and 1000 °C, respectively, for 60 s. For the pristine graphene, the G peak appeared at ∼1588 cm^–1^, and the 2D peak at ∼2698 cm^–1^. Meanwhile, for the nitrogen-doped graphene, the G peak appeared at ∼1595 cm^–1^, and the 2D peak at ∼2724 cm^–1^; both the G-peak and 2D peak exhibited blue shifts of approximately 7 cm^–1^ and 6–7 cm^–1^, respectively.

**Fig. 2:**
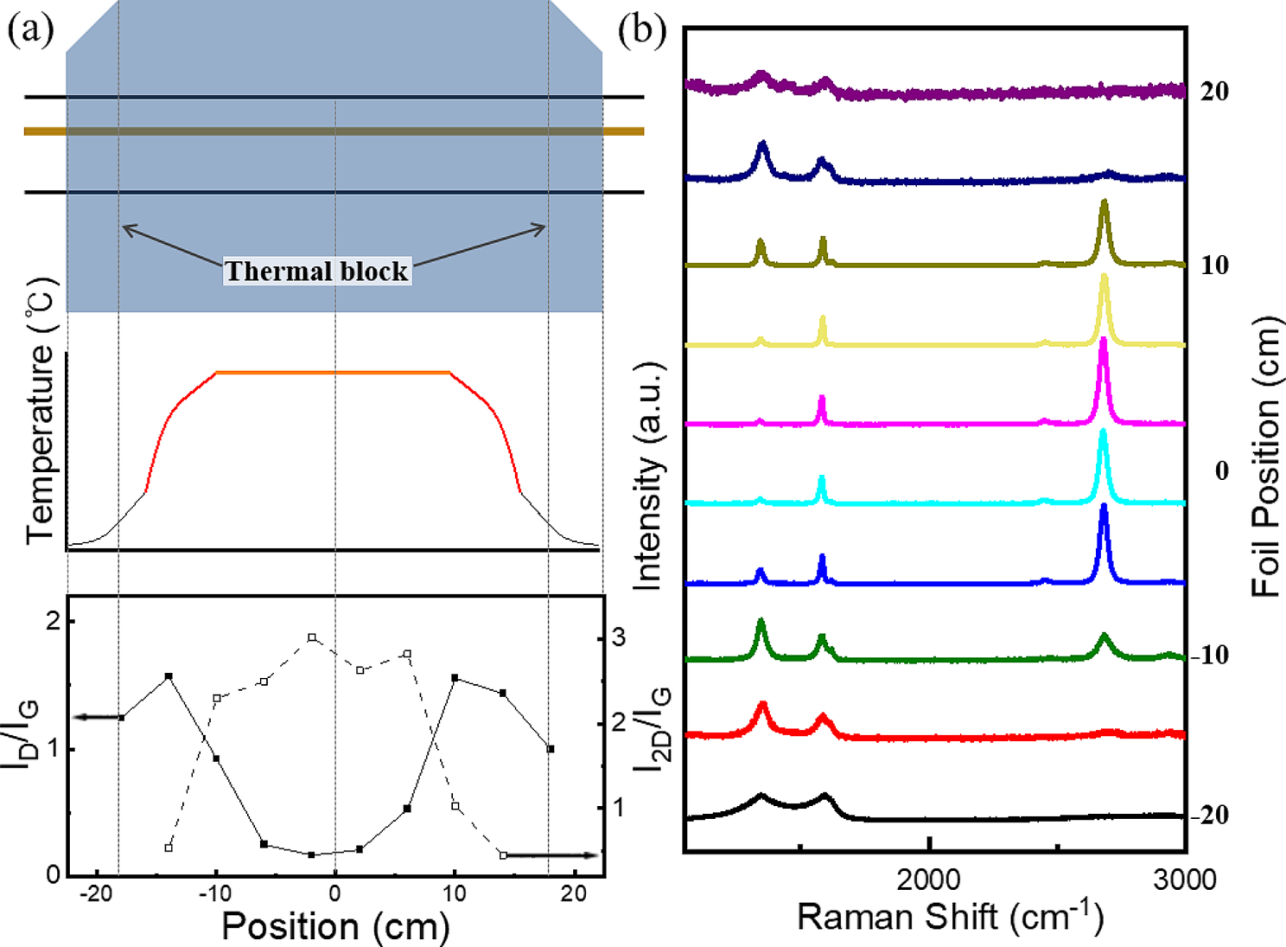
(**a**) Thermal profile of CVD furnace and (**b**) Raman spectra of graphene synthesized at various furnace positions

To determine the maximum graphene area achievable through conveyor-type CVD synthesis, graphene samples were synthesized at various positions along the furnace; the temperature was measured at each position, and the synthesized graphene samples were analyzed using Raman spectroscopy (Fig. 2). Using a 45 cm furnace as the reference, we confirmed the synthesis of high-quality graphene in an area measuring 5 cm × 15 cm, approximately equal to the size of a smartphone screen.

**Fig. 3:**
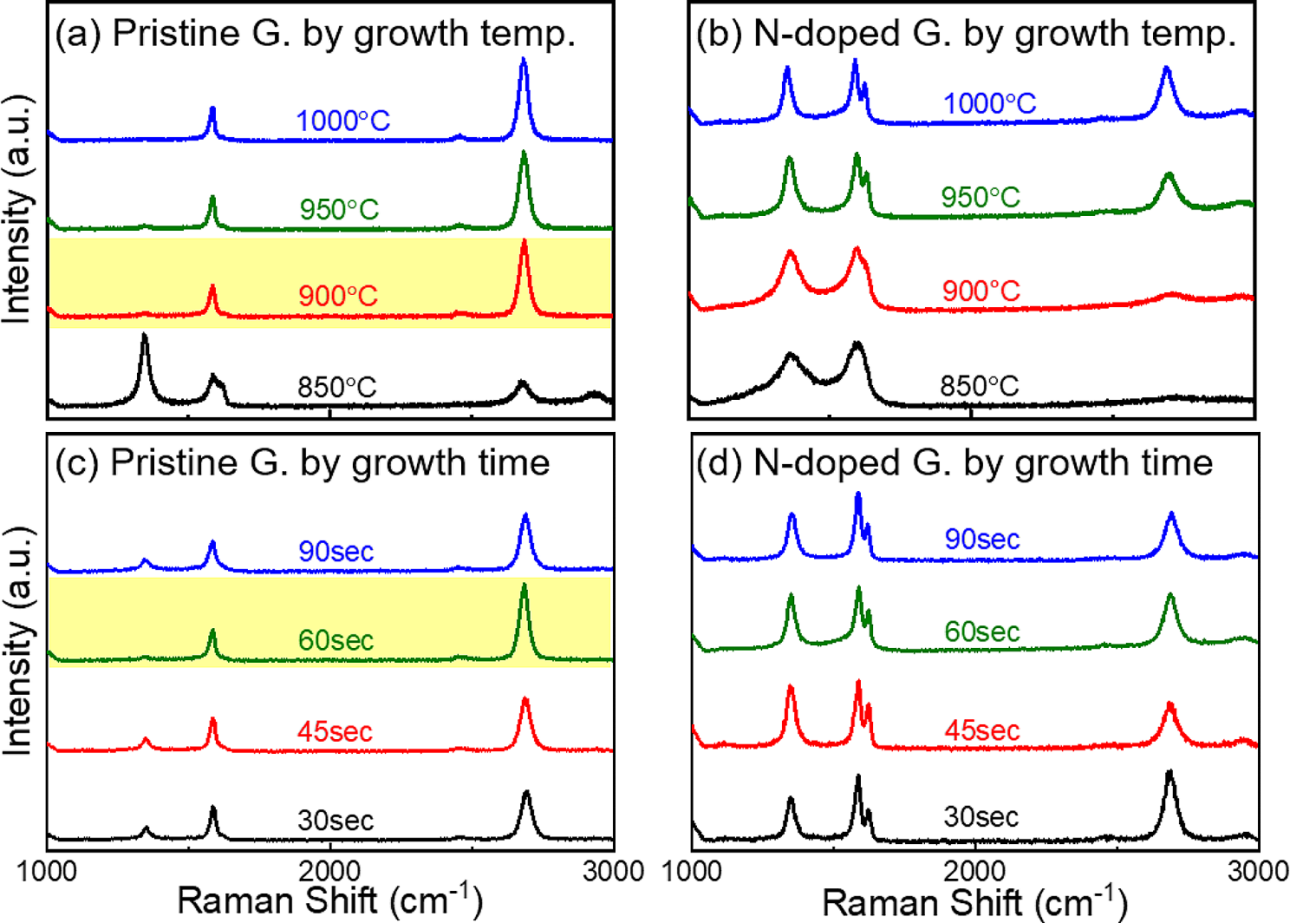
Growth temperature dependence Raman spectra of (**a**) pristine and (**b**) N-doped graphene. Growth time dependence Raman spectra of (**c**) pristine and (**d**) N-doped graphene

Figure 3 shows the Raman spectra of pristine and nitrogen-doped graphene synthesized using butane and pyridine as precursors, respectively, and transferred onto SiO_2_/Si substrates. Figure 3a shows the Raman spectra for pristine graphene synthesized using butane at varying temperatures of 850, 900, 950, and 1000 ℃ for 60 s, while Fig. 3c shows the Raman spectra for pristine graphene synthesized at 900 ℃ for varying growth times of 30, 45, 60, 90 s. Figure 3b and d show the corresponding data for nitrogen-doped graphene synthesized under the same conditions using pyridine as the precursor. Notably, the high-speed synthesis conditions afforded both pristine and doped graphene in just 60 s at a growth temperature of 900 ℃, and were applicable even when using a Si substrate with a thin copper film deposited on top (300 nm Cu/SiO_2_/Si). This demonstrated the suitability of these conditions for device fabrication processes (Figure S1).

As shown in Fig. 3a, pristine graphene samples synthesized at temperatures above 900 ℃ exhibited almost identical characteristics to that synthesized at 900 °C, indicating the high quality of the synthesized monolayer of pristine graphene. For N-doped graphene synthesized under the same conditions, the D/G ratio decreased and the 2D/G ratio increased with increasing temperature (Fig. 3c). While there is no clear relationship between synthesis time and Raman data for pristine graphene at 900 ℃ and N-doped graphene at 1000 ℃ (Fig. 2c, d), examining the peak ratio based on synthesis time for pristine graphene reveals that a low D/G ratio and high 2D/G ratio were achieved only at a synthesis time of 60 s, indicating the synthesis of high-quality and uniform graphene with small error bars (Figure S2). Considering cost and energy efficiency, as well as the potential for mass production and industrial competitiveness, the most suitable conditions for the synthesis of pristine graphene are 900 ℃ and 60 s. For nitrogen-doped graphene, the conditions at 1000 ℃ resulted in the highest 2D peak. However, the optimal conditions must be adjusted based on the required qualities and application purpose; this can be achieved by combining XPS and electrical property evaluation, considering factors such as the nitrogen content and charge neutrality point (Dirac point).

Based on the Raman data, additional analyses were conducted on the graphene samples synthesized at 900, 950, and 1000 ℃ for 60 s. For pristine graphene, the analysis was focused on the sample synthesized at the lowest temperature, 900 ℃, as the results did not differ significantly with varying temperatures. In other words, when the growth temperature was sufficiently high and the characteristics were saturated, there was minimal variation in the samples with temperature.

**Fig. 4:**
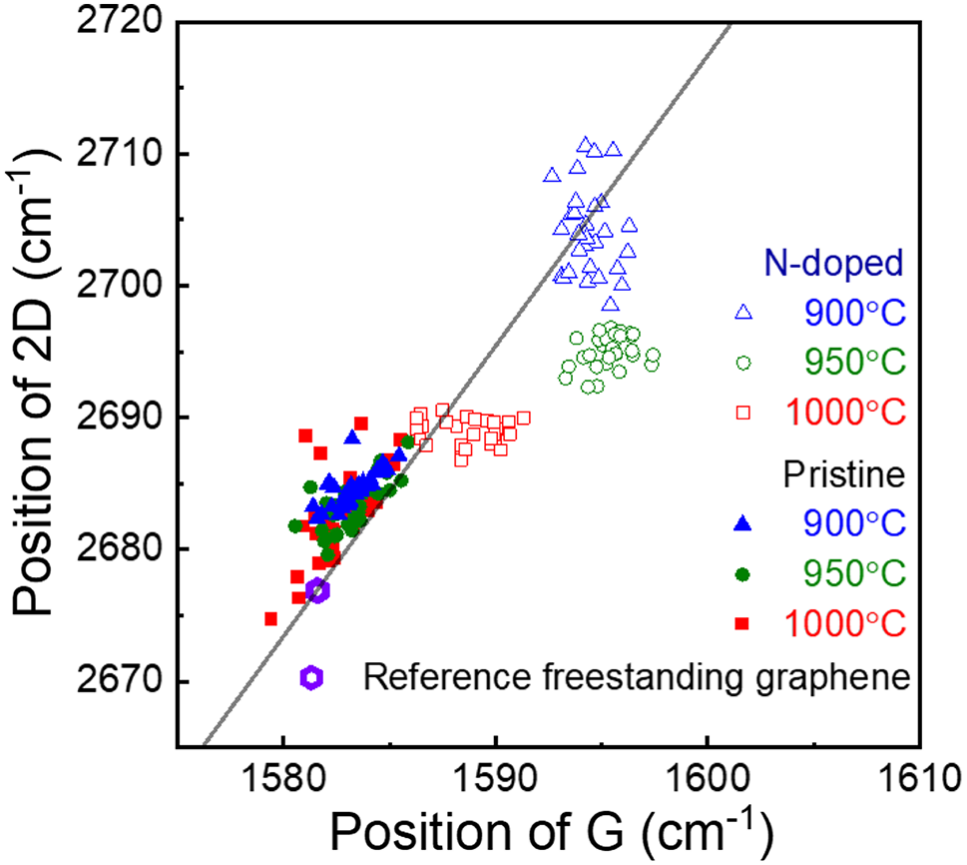
Correlation between the positions of the G and 2D Raman modes of graphene. The data were obtained from the Raman mapping of six graphene samples at 30 spots each. The purple dots represent the results for a freestanding graphene sample

In Fig. 4, the G peaks and 2D peaks for pristine and doped graphene synthesized at 900, 950, and 1000 ℃ were compared using Raman data measured at 30 spots for each sample. For pristine graphene, the G peak and 2D peak were located at approximately 1583 and 2684 cm^–1^, respectively, independent of growth temperature. Meanwhile, for doped graphene, at growth temperatures of 900, 950, 1000 ℃, the G peak was located at approximately 1595, 1594, and 1589 cm^–1^, respectively, and the 2D peak at approximately 2704, 2694, and 2688 cm^–1^, respectively. The purple dots represent the reference values for exfoliated freestanding graphene unaffected by strain or doping (G peak: 1581.6 ± 0.2 cm^–1^, 2D peak: 2676.9 ± 0.7 cm^–1^) [23]. The gray lines indicate the Raman changes due to strain, with a slope of 2.2 ± 0.2 (Pos(2D)/Pos(G)) [23]. For graphene synthesized using butane, three distinct graphene clusters were formed near the reference points, indicating uniform pristine graphene.

According to previous research, hole doping causes Raman spectra to exhibit a linear shift toward higher frequencies at a slope of 0.70 ± 0.05 (Pos(2D)/Pos(G)) and blue shifts toward higher frequencies [29]. However, studies on the effect of electron doping on the Raman spectra are relatively limited, though they have been reported as nonlinear shifts [30, 31]. Additionally, a previous study utilized freestanding graphene in top-gate transistors, making it challenging to directly compare and explain our Raman results for nitrogen-doped graphene, in which the dopant replaced carbon. In case of N-doped graphene using pyridine, both the G and 2D peaks exhibited a blue shift and nonlinear changes with decreasing synthesis temperature.

### X-Ray photoelectron spectroscopy

**Fig. 5:**
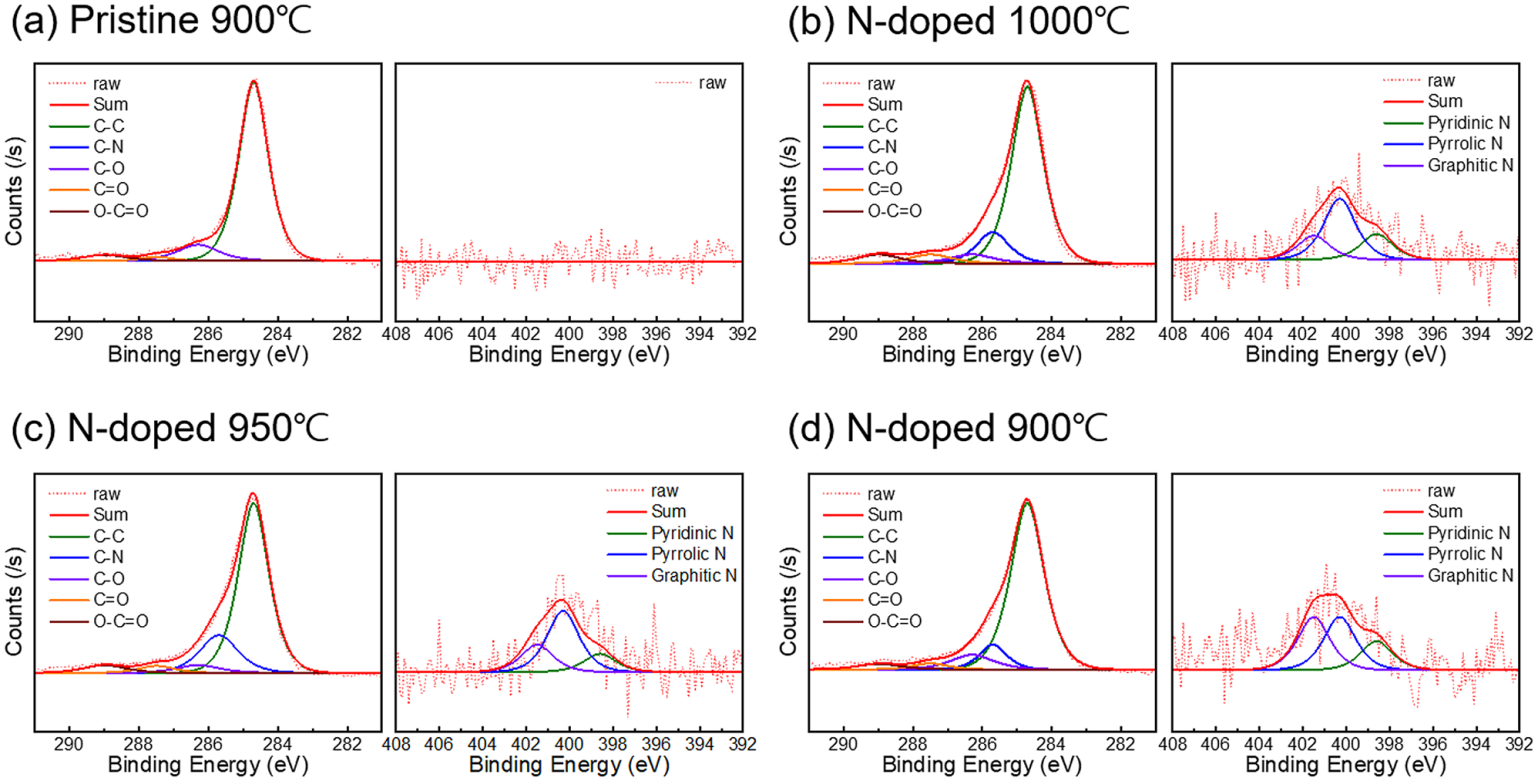
XPS C1s spectra (left) and N1s spectra (right) of (**a**) pristine graphene synthesized at 1000 ℃ and N-doped graphene synthesized at (**b**) 1000 ℃, (**c**) 950 ℃, (**d**) and 900 ℃. The C 1s peak can be split into five peaks at 284.7, 285.7, 286.3, 287.5, and 289.0 eV, which correspond to and are labeled as C–C (green), C–N (blue), C–O (purple), C = O (orange), and O–C = O (brown), respectively. The N 1s peak can be split into three peaks at 398.6, 400.3, and 401.5 eV, which correspond to and are labeled as pyridinic N (green), pyrrolic N (blue), and graphitic N (purple), respectively

Figure 5 shows the XPS data obtained for the elemental analysis of each graphene sample. Figure 5a shows the XPS spectrum of pristine graphene synthesized at 900 ℃, while Fig. 5b–d show the XPS spectra of nitrogen-doped graphene synthesized at 1000, 950, and 900 ℃, respectively. The C1s spectra for both graphene types include a dominant peak at 284.7 eV corresponding to the C–C bond, a small oxide bond that can occur during the process at 286.3 eV corresponding to the C–O bond, and peaks at 287.5 and 289.0 eV corresponding to the O–C = O and C = O bonds, respectively [13–15, 20, 32–34] For nitrogen-doped graphene, there was an additional clear peak corresponding to the C–N bond at 285.7 eV, and all C1s peaks exhibited overall broadening. The N1s spectra can comprise three main peaks corresponding to three different bonds: the pyridinic N bond (398.6 eV) and pyrrolic N bond (400.3 eV) represent the bonding of one and two nitrogen atoms, respectively, with carbon through π bonds, and the graphitic N bond at 401.5 eV corresponds to the substitution of carbon with nitrogen [13, 14]. Although all three bonds involve nitrogen and carbon, pyridinic N and pyrrolic N contribute to p-type doping, whereas graphitic N contributes to n-type doping [32–34]. These peaks are not present for pristine graphene, indicating the absence of nitrogen. Meanwhile, all doped graphene samples were confirmed to contain nitrogen owing to the presence of all three peaks, with the nitrogen content being similar independent of the synthesis temperature; however, as the synthesis temperature decreased from 1000 to 900 ℃, the ratio of graphitic-N increased.

### Electrical properties

**Fig. 6:**
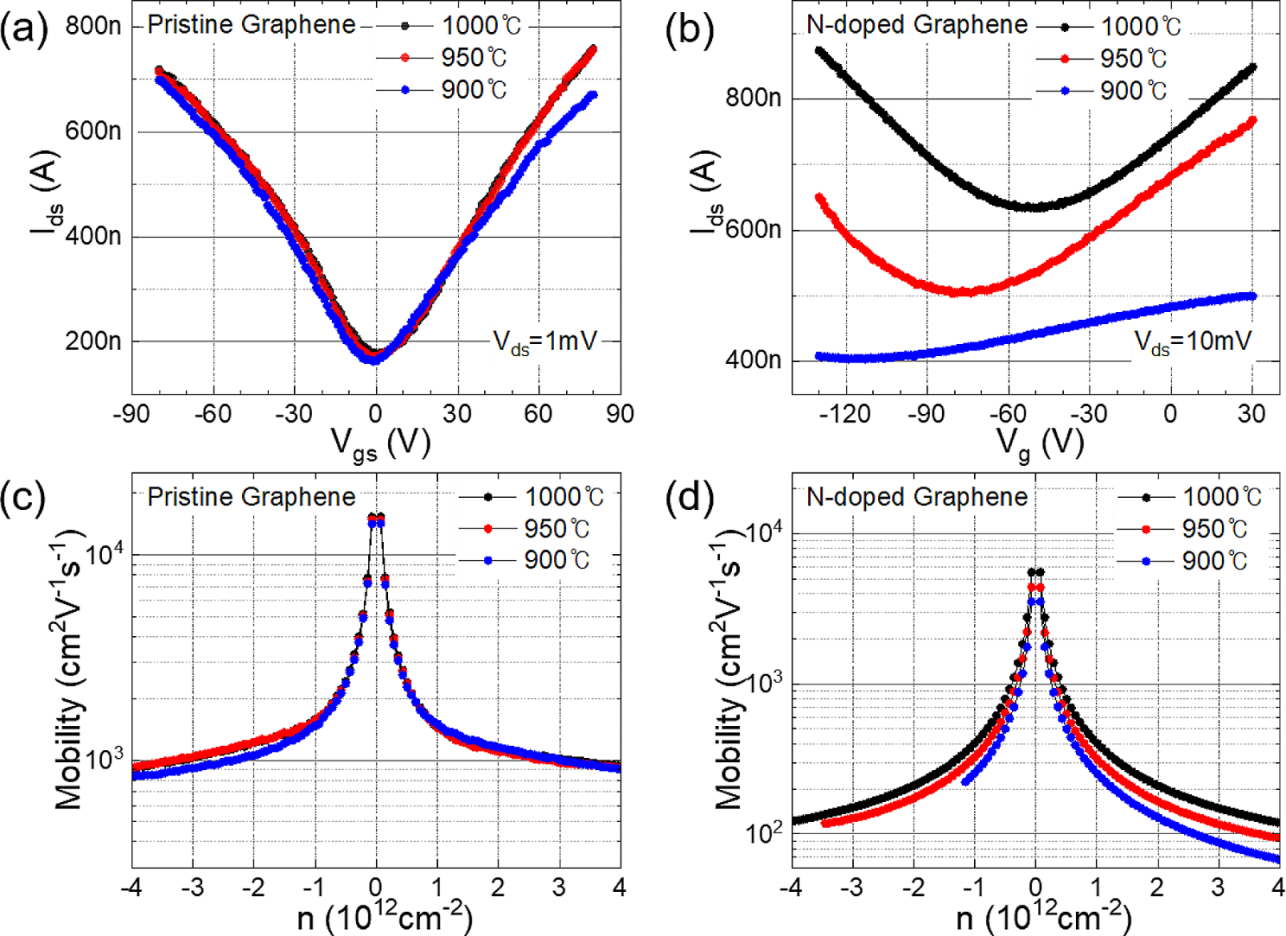
Electrical properties of (**a**,**c**) pristine and (**b**,**d**) N-doped graphene. (**a**,**b**) I_DS_–V_GS_ characteristic of pristine graphene synthesized at various growth temperatures and (**c**,**d**) carrier mobilities calculated from (**a**)

Figure 6a shows the transfer curve of pristine graphene with respect to the growth temperature. Pristine graphene synthesized at three different temperatures exhibited Dirac points ranging from 0 to + 1 V, and the values of the source-drain current with respect to the gate voltage were similar. As observed from the Raman data, the electrical properties of pristine graphene were nearly identical when synthesized at growth temperatures above 900 ℃. This is consistent with Fig. 5(a), in which the absence of C–N and N1s is logically presented. The electron mobility was calculated [21] to be 1430–1498 cm^2^ V^–1^ s^–1^ at a charge concentration of n = + 1e^12^, and the hole mobility was calculated to be 1468–1572 cm^2^ V^–1^ s^–1^ at *n* = − 1e^12^.

In the case of N-doped graphene, the Dirac point voltage was consistently below 0 V, indicating n-type behavior. For graphene samples synthesized at 900, 950, and 1000 °C, the Dirac point voltages were − 117, − 82, and − 51 V, respectively, electron mobilities at n = + 1e^12^ were calculated as 253, 317, and 402 cm^2^ V^–1^ s^–1^, respectively, and hole mobilities at *n* = − 1e^12^ were calculated as 255, 326, and 404 cm^2^ V^–1^ s^–1^, respectively. As the growth temperature decreased, the relative abundance of graphitic N increased, leading to a larger absolute value of the Dirac point voltage in the negative direction. However, overall, the graphitic structure decreased, and the increased scattering sites resulted in a reduction in mobility. The mobilities of graphene were measured on 15 devices for each sample, and the average properties and distribution by Dirac point voltage and mobilities are shown in Figure S4.

### Gas sensing application

Gas reaction of graphene is similar to carbon nanotubes(CNTs). According to the research studied the reaction of carbon nanotubes to NO_2_ and NH_3_, Single-walled CNTs is insensitive to both gases than multi-walled or doped CNTs because of metallic structure [35]. Moreover, n-type doping functionalizes CNTs electron rich, leads it being sensitive to NO_2_ which withdraws electrons while being insensitive to NH_3_ [36]. It leads to explain the difference in the reactivity of n-type graphene to the two gases. In the case of graphene, results were reported showing better sensitivity of doped graphene compared to pristine graphene, regardless of n-type [37] or p-type [38]. Our application of graphene as a gas sensor is consistent with the existing researches have reported.

**Fig. 7:**
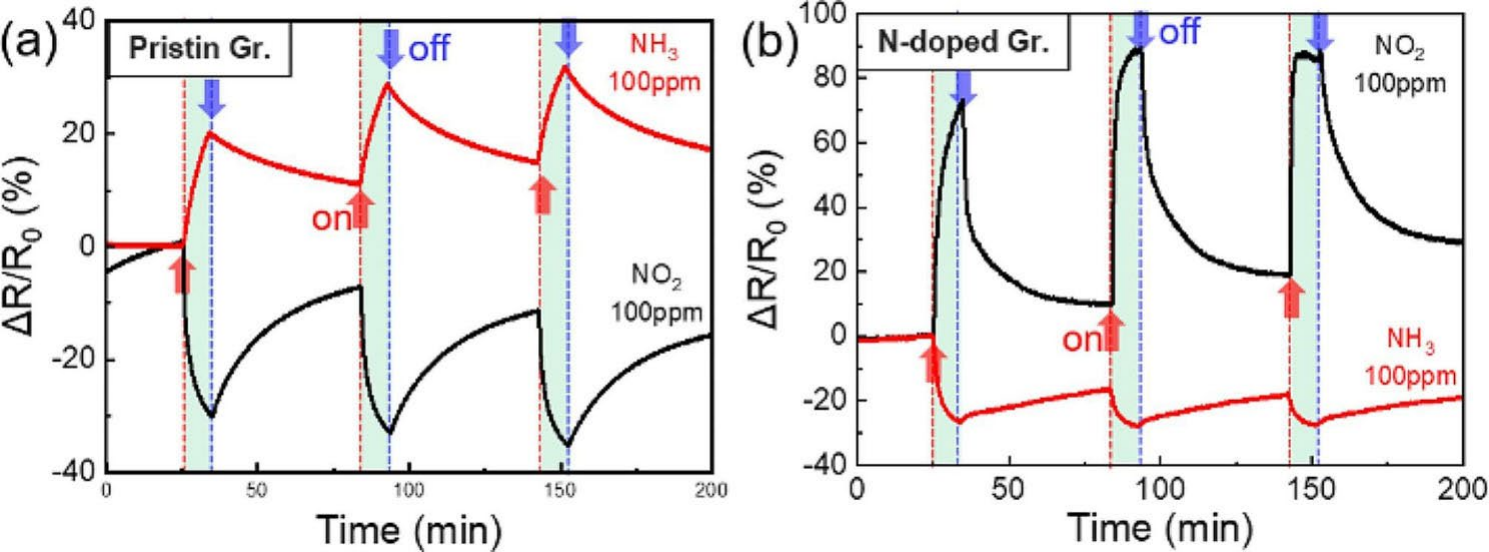
NO_2_ and NH_3_ gas response characteristics of (**a**) pristine graphene and (**b**) N-doped graphene

Figure 7 shows the potential applicability of gas sensors using pristine graphene synthesized at 900 ℃ and N-doped graphene synthesized at 1000 ℃. The two types of graphene were transferred onto SiO_2_ substrates to create simple FET devices, which were then applied as gas-sensor channels for NO_2_ and NH_3_ at room temperature and atmospheric pressure. Typically, CVD-grown graphene, the dominant carriers are holes, is a p-type channel; NO_2_ absorbed onto the graphene surface withdraws free electrons such that the resistance increases, while NH_3_ donates free electrons to the graphene surface, thus the resistance decreases. Meanwhile, nitrogen-doped graphene, the dominant carriers are electrons, is an n-type channel that exhibits the opposite electrical response characteristics. This leads to opposite changes in the reaction characteristics.

**Fig. 8:**
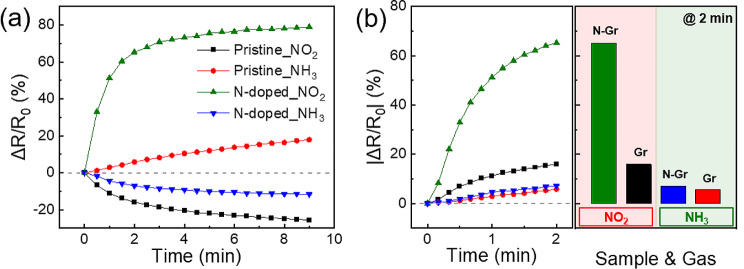
(**a**) NO_2_ and NH_3_ gas response characteristics of pristine and N-doped graphene for one cycle and (**b**) magnified absolute sensitivity for 2 min

The graphene-based gas sensors were exposed to NO_2_ and NH_3_ gases for 9 min each, and the resistance changes were measured repeatedly. Pristine graphene exhibited a polarity characteristic similar to that of a previous study, [39] showing adsorption and desorption based on the gas type. In contrast, the N-doped graphene exhibited reversed polarity. Figure 8a shows the second cycle of the graphs in Fig. 7, grouped according to graphene and gas types. Pristine and N-doped graphene exhibited reactivities of − 25% and 79%, respectively, when exposed to NO_2_ for 9 min, and reactivities of 18% and − 12%, respectively, when exposed to NH_3_ for 9 min. Figure 8b shows the reactivity of the graphene samples after 2 min of exposure to each gas; nitrogen-doped graphene exhibited higher sensitivity and faster response compared to pristine graphene for both gases.

## Conclusion

To enhance the potential for the industrial synthesis of graphene, we developed a conveyor CVD system for the highly productive synthesis of high-quality graphene. By optimizing the synthesis conditions, we successfully synthesized pristine and nitrogen-doped graphene using the liquid precursors butane and pyridine, respectively; 12 samples were synthesized in 1 h, confirming the potential for rapid synthesis. This system not only allows for the simultaneous synthesis of numerous graphene samples, but enables the synthesis of graphene with the desired qualities by using various precursors and different recipes. In addition, it can accommodate flexible foils, rigid substrates, and powder catalysts for graphene synthesis.

At synthesis temperatures above 900 °C, we achieved high quality pristine graphene samples that exhibited low D/G and 2D/G ratios in their Raman spectra. XPS analysis indicated dominant C–C peaks, excluding oxides formed during the transfer process, and no N1s peaks. The Dirac point voltage ranged from 0 to 5 V, exhibiting characteristics of neutral CVD graphene. Meanwhile, for the nitrogen-doped graphene samples, decreasing the growth temperature blue-shifted the G-peak and 2D-peak in the Raman spectra, increased the ratio of graphitic-N in the XPS spectra, and decreased the Dirac point voltage and mobility with a unidirectional tendency. Therefore, for future research and development applications, synthesis closer to 900 °C may be preferred for synthesizing graphene with a higher level of nitrogen doping, while synthesis closer to 1000 °C can be selectively employed if graphene with lower nitrogen doping and better mobility is needed based on the specific objectives.

### Electronic supplementary material

Below is the link to the electronic supplementary material.

Supplementary Material 1

Supplementary Material 2

## Data Availability

The datasets used during the current study are available from the corresponding author on reasonable request.
