# Supplementary material for: Conveyor CVD to high-quality and productivity of large-area graphene and its potentiality
Source: Nano Converg. 2024 Aug 14;11:32. doi: 10.1186/s40580-024-00439-0 (PMC11324640; doi:10.1186/s40580-024-00439-0)
Supplement: Supplementary file 1 — Supplementary Material 1 [file 40580_2024_439_MOESM1_ESM.docx]

**Supporting information for**

**Conveyor CVD to high-quality and productivity of large-area graphene and its potentiality**

Dong Yun Lee^1^, Jungtae Nam^1^, Gil Yong Lee^1^, Imbok Lee^1^, A-Rang Jang^2, *^, and Keun Soo Kim^1, *^

^1^ Department of Physics and Graphene Research Institute, Sejong University, Seoul, 05006, Republic of Korea

^2^ Division of Electrical, Electronic and Control Engineering, Kongju National University, Cheonan-si, Chungcheongnam-do, 31080, Republic of Korea

^*^ indicates the corresponding author

^*^ Corresponding author: Keun Soo Kim^1^, A-Rang Jang^2^

Tel.: +82-02-3408-39881, +82-041-521-91872

E-mail address: kskim2676@sejong.ac.kr^1^, arjang@kongju.ac.kr^2^


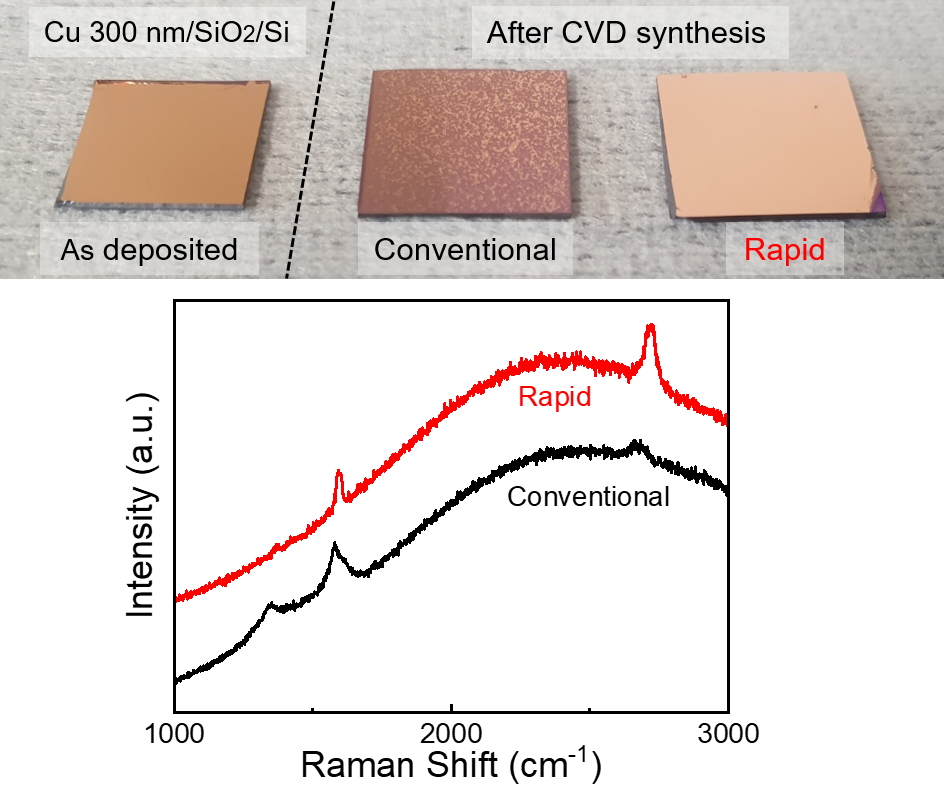


**Figure S1.** Comparison of photograph images for thermally deposited copper film(300 nm) on SiO_2_/Si substrate and conventional/rapid synthesis of graphene on it and their Raman spectra.


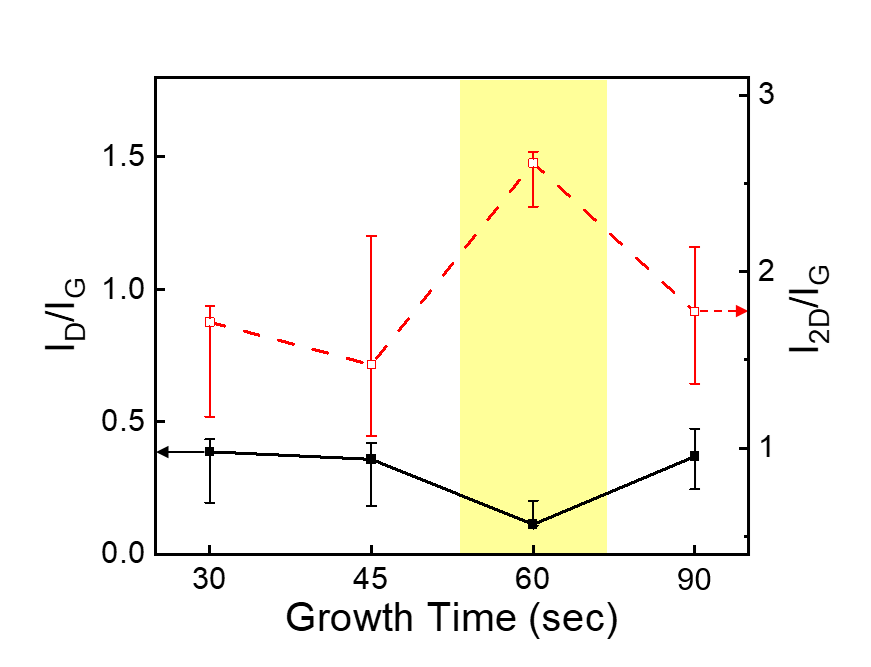


**Figure S2.** Raman peak ratio of pristine graphene synthesized at 900 ℃ by growth time. Compared to 60 sec data, 30, 45 and 90 sec data have larger D peak and smaller 2D peak, and bigger error bars indicate that the properties of each graphene are non-uniform.


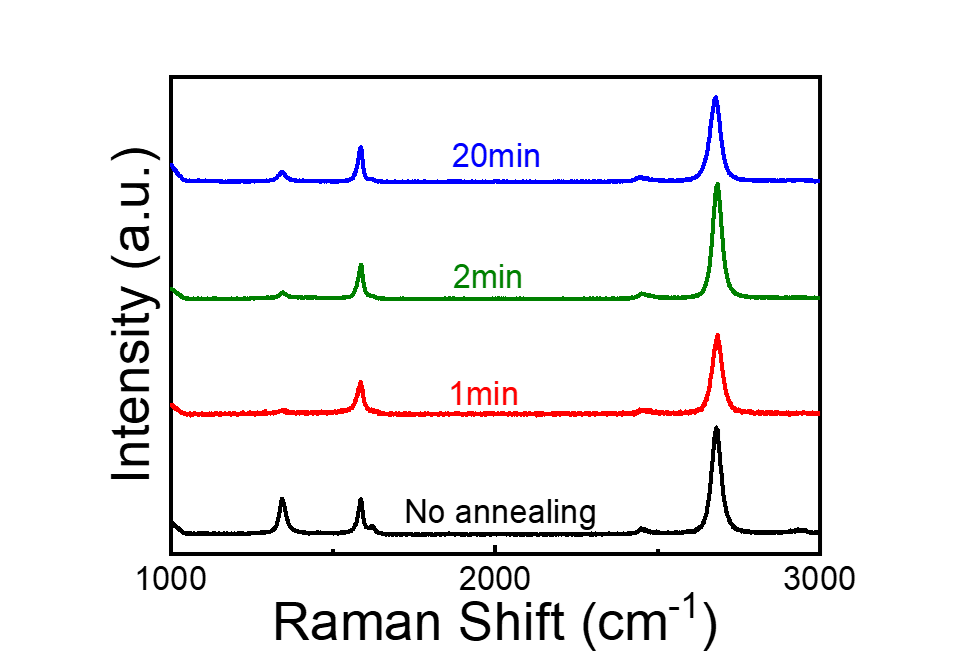


**Figure S3.** Annealing time dependence Raman spectra of pristine graphene at 1000 ℃.


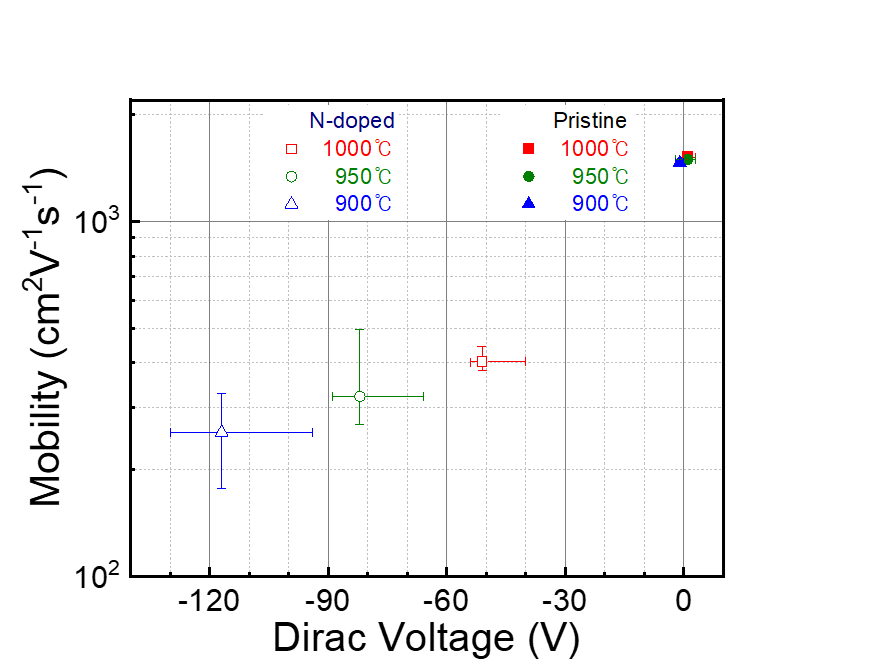


**Figure S4.** Distribution of carrier motilities and Dirac point in pristine and N-doped graphene depend on synthesis temperature.


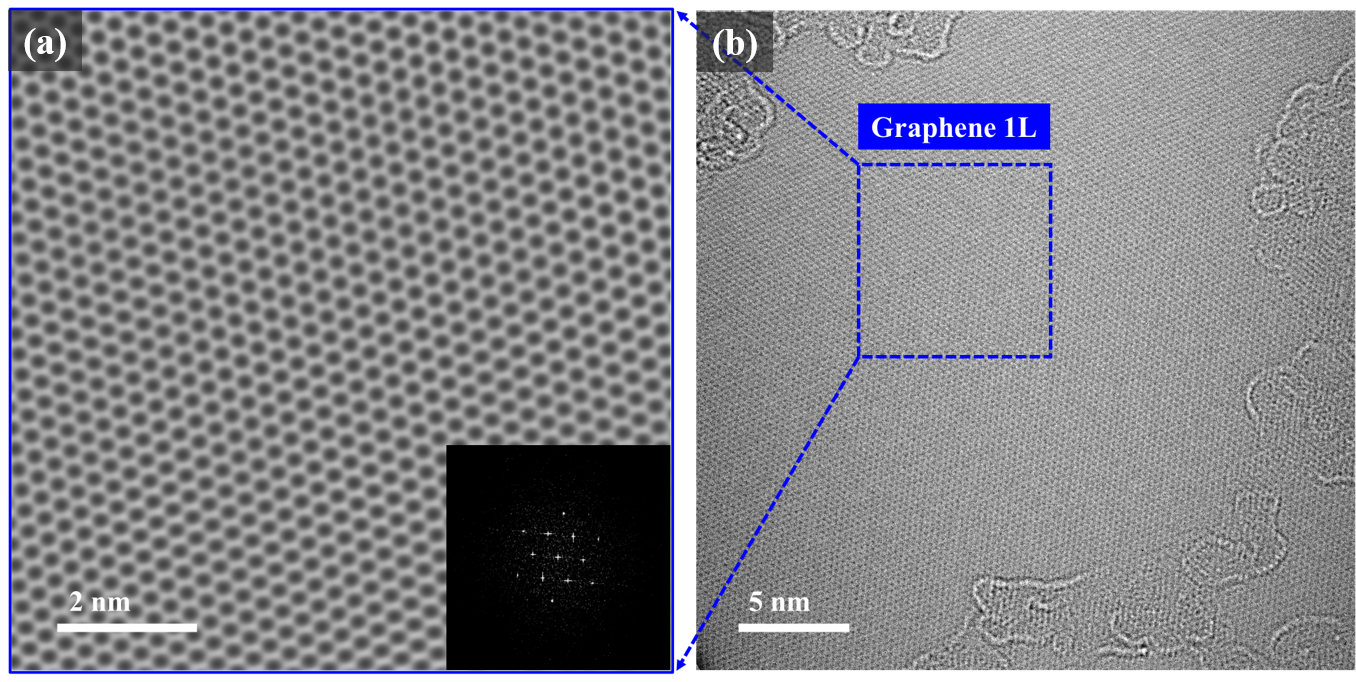


**Figure S5.** TEM images of pristine graphene. (a) HR-TEM image (inset; SAED pattern) from blue-square area of (b), (b) Low magnification TEM image.
